# Sex hormone–psoriasis interactions: from sex-specific clinical features to immunoregulatory mechanisms

**DOI:** 10.3389/fimmu.2026.1731024

**Published:** 2026-02-06

**Authors:** Yingying Dai, Yuanting Wang, Yuwei Huang, Xian Jiang, Jing Tan

**Affiliations:** 1 Department of Dermatology, West China Hospital, Sichuan University, Chengdu, China; 2 Laboratory of Dermatology, Clinical Institute of Inflammation and Immunology, Frontiers Science Center for Disease-related Molecular Network, West China Hospital, Sichuan University, Chengdu, China; 3 General Practice Medical Center, Clinical Epidemiology and Evidence-based Medicine Center, West China Hospital, Sichuan University, Chengdu, China; 4 NHC Key Laboratory of Clinical Epidemiology and Evidence-based Medicine, West China Hospital, Sichuan University, Chengdu, China

**Keywords:** immune inflammation, immune-endocrine, psoriasis, sex differences, sex hormones

## Abstract

Psoriasis is a chronic inflammatory skin disease mediated by T cells, characterized by distinct sex differences and variations across different reproductive stages, suggesting that sex hormones may play a significant role in its pathogenesis. In recent years, research has increasingly focused on the bidirectional effects of sex hormones in psoriasis: on one hand, changes in hormone levels can affect the onset and progression of psoriasis; on the other hand, the systemic inflammation of psoriasis can interfere with the homeostasis of the sex hormone axis. This review systematically integrates clinical epidemiological evidence of sex hormone abnormalities in cutaneous psoriasis patients, outlines the molecular mechanisms of estrogen, androgen, and progesterone in immune-inflammatory regulation of psoriasis, and further explores how psoriasis-related inflammation, through cytokines, stress responses, and metabolic abnormalities, can in turn disrupt sex hormone balance. The focus is primarily on immunopathological mechanisms, with a secondary consideration of the impact of metabolic and stress-related factors, which modulate immune responses and may indirectly influence disease progression. We highlight the complex immune-endocrine network interaction between sex hormones and cutaneous psoriasis, emphasizing the need for future sex-stratified studies, dynamic hormone monitoring, and mechanistic validation models to clarify their causal pathways. This will aid in understanding the sex-specific clinical manifestations of psoriasis and provide a theoretical basis for developing hormone-targeted intervention strategies.

## 1 Introduction

Psoriasis is a chronic, relapsing, immune-mediated inflammatory skin disease with a global prevalence of approximately 1–3%, affecting over 125 million individuals (1, 2). It is characterized by erythema, silvery-white scales, and chronic inflammation, which make it the second leading contributor to skin-related disability (3). The pathogenesis of psoriasis is complex, involving an immune-inflammatory network centered on Interleukin (IL)-17 and IL-23, aberrant keratinocyte proliferation and differentiation, skin barrier dysfunction, and genetic susceptibility (4). While psoriasis can manifest systemically, including as psoriatic arthritis, this review primarily focuses on its cutaneous manifestations, with an emphasis on the immunopathological mechanisms involved in the skin-endocrine interface. Epidemiological studies indicate that the overall prevalence of psoriasis is similar between males and females, yet significant sex differences exist in disease presentation, severity, and clinical course (5, 6). Female patients typically experience an earlier age of onset and milder disease severity, whereas male patients are more likely to develop severe psoriasis and have higher rates of systemic treatment use (7, 8). Notably, female patients exhibit characteristic fluctuations in cutaneous psoriasis symptoms across different reproductive stages—including the menstrual cycle, pregnancy, postpartum period, and perimenopause (9). Higher estrogen levels are associated with symptom improvement, while the sharp decline in hormone levels during perimenopause often coincides with increased disease activity, suggesting that sex hormones may serve as key regulatory factors in psoriasis (10).

The regulatory role of sex hormones in various autoimmune diseases, including systemic lupus erythematosus, multiple sclerosis, and rheumatoid arthritis, has been well established (11). On the one hand, sex hormones modulate immune cell function through genomic and non-genomic mechanisms, with their effects depending not only on ligand concentration but also on the density, distribution, and downstream signaling of corresponding receptors (12). On the other hand, the chronic inflammatory state of psoriasis can activate the hypothalamic-pituitary-adrenal (HPA) axis, upregulating the secretion of pro-inflammatory cytokines such as IL-17, which in turn disrupts the hypothalamic-pituitary-gonadal (HPG) axis and subsequently impacts sex hormone levels (13–15). While these interactions are important, this review primarily focuses on immune-inflammatory regulation through sex hormones, with the HPA/HPG axis and metabolic factors considered as supplementary influences. Although existing studies suggest a complex interplay between sex hormones and psoriasis, the molecular mechanisms underlying the role of specific sex hormones in the immune-inflammatory response of psoriasis remain unclear. This review aims to systematically evaluate the clinical evidence linking sex hormones to cutaneous psoriasis, elucidate the potential mechanisms of their bidirectional interactions at the skin-endocrine interface, and provide a theoretical foundation for the development of sex-specific therapeutic strategies in the context of precision medicine.

## 2 Clinical evidence of sex hormone dysregulation in psoriasis patients

### 2.1 Sex hormone characteristics in female psoriasis patients

Female psoriasis patients exhibit characteristic clinical patterns associated with sex hormones, with disease onset and progression closely linked to hormonal fluctuations (**Table 1**). Epidemiological studies indicate that female psoriasis presents a bimodal onset pattern, peaking in late puberty and perimenopause, with the disease course significantly modulated by physiological states such as menstruation, pregnancy, and menopause, suggesting that estrogen and other female hormones may play a critical role in the pathogenesis of psoriatic skin inflammation (16–18).

**Table 1:** Clinical and hormonal characteristics of female psoriasis patients across studies.

Study design	Author, publication year	Sample size	Sex hormones related	Evidence in the literature
Prospective cohort study	Yang, 2025 (26)	n1 = 2, 796, 101 (>50years), ncase1 = 24, 694; n2 = 5, 435, 182 (≤50years), ncase2 = 46, 430	E, P	Hormone therapy (HT) was associated with an increased risk of psoriasis, with a higher risk observed in younger women. Among women aged >50 years, the hazard ratio (HR) was 1.48 (95% CI: 1.44–1.52) in the intention-to-treat (ITT) analysis and 5.93 (95% CI: 5.66–6.22) in the per-protocol (PP) analysis. Among women aged ≤50 years, the HR was 1.93 (95% CI: 1.90–1.99) in the ITT analysis and 7.85 (95% CI: 7.56–8.15) in the PP analysis.
Prospective cohort study	Go, 2023 (25)	n=1, 130, 741, ncase=36, 286	E, P	Use of hormone replacement therapy (HRT) for ≥5 years was associated with a significantly increased risk of psoriasis (HR = 1.22, 95% CI: 1.16–1.29); increased risks were also observed in the <2 years group (HR = 1.13, 95% CI: 1.09–1.17) and the 2–5 years group (HR = 1.21, 95% CI: 1.15–1.26). Incidence rates increased with longer duration of HRT use (no HRT: 3.36 per 1, 000 person-years; ≥5 years: 4.09 per 1, 000 person-years).
Prospective cohort study	Xiao, 2024 (24)	n1 = 139, 572, ncase1 = 1, 253(PsO); n2 = 142, 329, ncase2 = 301 (PsA)	E	A later age at natural menopause (ANM) and a longer reproductive lifespan (RYs) were associated with a dose–response reduction in the risk of late-onset psoriasis and psoriatic arthritis (PsA). Compared with ANM <45 years, ANM ≥55 years was associated with a 34% lower risk of psoriasis (HR = 0.66, 95% CI: 0.53–0.82) and a 46% lower risk of PsA (HR = 0.54, 95% CI: 0.35–0.84). Compared with RYs <38 years, RYs ≥38 years were associated with a 23% lower risk of psoriasis (HR = 0.77, 95% CI: 0.69–0.87) and a 34% lower risk of PsA (HR = 0.66, 95% CI: 0.52–0.84).
Prospective cohort study	Wu, 2016 (23)	n=163, 763, ncase=1, 253	E2, P	A persistently irregular menstrual cycle during adulthood (HR = 1.32, 95% CI: 1.01–1.73) and surgical menopause (HR = 1.19, 95% CI: 1.01–1.40) were associated with a modestly increased risk of psoriasis; current use of hormone therapy was associated with a slightly elevated risk (HR = 1.22, 95% CI: 0.99–1.50), which was not statistically significant.
Prospective cohort study	Vessey, 2000 (147)	n=17, 032, ncase=92	E2, P	Current or recent users of oral contraceptives exhibited a relative risk for psoriasis of RR = 1.7, which was not statistically significant, and showed no association with duration of use or time since last use.
Prospective cohort study	Bello, 2022 (20)	n=169, ncase=82	E, P	Among patients with psoriasis, no significant changes were observed in disease severity, pruritus, or quality of life across different phases of the menstrual cycle; gynecological history (including age at menarche, menstrual cycle length, and oral contraceptive use) was not associated with symptom variation.
Prospective cohort study	Murase, 2005 (22)	n=74, ncase=47	E2, E3, E1, P	During pregnancy, 55% of patients experienced improvement in psoriasis, with serum E2, E3, and the estrogen-to-progesterone ratio showing significant or near-significant associations with BSA improvement, whereas progesterone alone showed no association.
Secondary data analysis	Chan, 2021 (27)	n=19, 311	E2, P	Use of estrogen alone (CEE) was not significantly associated with psoriasis risk (HR = 1.11, 95% CI: 0.82–1.50, *P* = 0.49), whereas combined estrogen–progestin therapy (CEE + MPA) was associated with a reduced risk (HR = 0.77, 95% CI: 0.60–0.98, *P* = 0.04).
Retrospective cohort study	Burle, 2025 (148)	ncase=71	E, P	During pregnancy, 52.2% of patients experienced disease relapse, 41.8% showed no change in skin lesions, and 4.5% demonstrated improvement. Discontinuation of biologic therapy prior to conception was associated with a significantly increased risk of psoriasis exacerbation (*p* < 0.02). Postpartum, 73% of patients experienced disease worsening.
Retrospective cohort study	McHugh, 1989 (149)	ncase=33	E, P	18% of patients developed psoriatic arthritis within three months postpartum, and 15% at menopause, suggesting that hormonal fluctuations may be a potential risk factor.
Case–control study	Dyulmesova-Bilash, 2021 (150)	ncase=100, ncontrol=30	FSH, LH, PRL, E2, P, T	Psoriasis patients with menstrual disorders exhibited a lower LH/FSH ratio than the normal range, elevated prolactin levels, and insufficient luteal-phase progesterone, while estradiol and testosterone levels remained within normal limits. Elevated FSH (>48 mIU/mL), elevated LH (>27 mIU/mL), reduced PRL (<348 ng/mL), reduced E2 (<21 pg/mL), and elevated T (>0.47 ng/mL) were associated with an increased risk of acute psoriasis.
Case–control study	Tuğrul Ayanoğlu, 2018 (151)	ncase=14, ncontrol=25	E2, FSH, LH	Patients with psoriasis exhibited higher levels of FSH (6.76 vs. 5.71 mIU/mL, p = 0.039) and FSH/LH ratio (1.52 vs. 0.92, p < 0.05), and a lower antral follicle count (AFC) (5 vs. 7, p < 0.05) compared with controls, suggesting diminished ovarian reserve; no significant differences were observed in E2, LH, or menstrual regularity.
Case	Stevens, 1993 (19)	ncase=1	E2	Psoriasis and psoriatic arthritis exhibited significant exacerbation during the ovulatory phase (day 14) and the premenstrual phase (day 26) of the menstrual cycle, and anti-estrogen therapies (tamoxifen and goserelin) were effective.
Case	Boyd, 1999 (145)	ncase=1	E2	Vulvar psoriasis lesions exacerbate two days before the onset of menstruation and ten days post-menstruation. Treatment with tamoxifen significantly alleviates itching and skin lesions.

The cyclic variations of estrogen and progesterone during the female menstrual cycle provide a natural observational window for studying the impact of hormones on psoriasis. An early case report by Stevens et al (19) described a typical case in which the severity of psoriatic arthritis was closely correlated with the menstrual cycle, with symptoms significantly worsening during the ovulatory and premenstrual phases, while anti-estrogen therapy (goserelin) demonstrated significant efficacy in treating this cyclic psoriatic arthritis. However, in a 3-month prospective study, Bello et al (20) continuously assessed psoriasis symptoms across the premenstrual, menstrual, and postmenstrual phases and found no significant correlation between psoriasis severity and the menstrual cycle, suggesting potential individual variability in the influence of the menstrual cycle on psoriasis.

Pregnancy provides critical evidence for studying the effects of prolonged high-level estrogen exposure on psoriasis. Clinical observations consistently indicate that psoriasis often improves or even achieves complete remission during pregnancy, but relapses following delivery as hormone levels sharply decline (21). A prospective cohort study further quantified this association: elevated serum estradiol levels (P = 0.009, r=0.648), increased estriol levels (P = 0.06, r=0.491), and a higher estrogen-to-progesterone ratio (P = 0.006, r=0.671) were positively correlated with significant improvements in psoriasis-affected body surface area, whereas changes in progesterone levels showed no significant association with psoriasis improvement (22). These findings suggest that estrogen plays a critical role in psoriasis remission.

Large-scale cohort studies provide robust evidence for the long-term protective effects of endogenous estrogen. Analyses from the Nurses’ Health Study I and II found that women with irregular menstruation (hazard ratio, HR = 1.32) and surgical menopause (HR = 1.19) had a significantly increased risk of psoriasis onset, suggesting that endogenous estrogen deficiency may enhance psoriasis susceptibility (23). In 2024, Xiao et al (24) confirmed through cohort studies and Mendelian randomization analyses that late menopause and longer reproductive lifespan were significantly associated with a dose-dependent reduction in late-onset psoriasis risk (P<0.001), further corroborating the protective effect of prolonged endogenous estrogen exposure on psoriasis.

On the other hand, the relationship between exogenous hormone replacement therapy (HRT) and psoriasis risk is complex. A nationwide cohort study involving 1.13 million postmenopausal women found that long-term HRT use (≥5 years) significantly increased the risk of psoriasis onset (HR = 1.22; 95% confidence interval, 95% CI: 1.16–1.29) (25). A Taiwanese cohort study, including 1, 482, 322 postmenopausal women and 3, 849, 721 women of reproductive age, further confirmed this finding: in postmenopausal women, the intention-to-treat analysis yielded a hazard ratio of 1.48 (95% CI: 1.44–1.52), while the per-protocol analysis showed a hazard ratio of 5.93 (95% CI: 5.66–6.22). Among women of reproductive age, the risk was even more pronounced, with an intention-to-treat analysis hazard ratio of 1.93 (95% CI: 1.90–1.99) and a per-protocol analysis hazard ratio of 7.85 (95% CI: 7.56–8.15) (26). Additionally, a secondary analysis of the Women’s Health Initiative randomized controlled trial indicated that estrogen-only therapy did not significantly increase psoriasis risk compared to placebo (HR = 1.11, 95% CI: 0.82–1.50, P = 0.49), whereas combined estrogen-progesterone therapy reduced psoriasis risk (HR = 0.77, 95% CI: 0.60–0.98, P = 0.04) (27). These differences may stem from variations in adjusted confounding variables and heterogeneity in study populations; for instance, the Taiwanese cohort study and Korean cohort studies did not adjust for key variables such as natural menopause age or reproductive years, potentially leading to a study population skewed toward women with early menopause (28). These findings suggest that different hormone combinations have varying effects on psoriasis risk, though the underlying mechanisms require further investigation.

### 2.2 Sex hormone characteristics in male psoriasis patients

Studies on sex hormone dysregulation in male psoriasis patients are relatively scarce. However, existing evidence confirms that male patients also exhibit characteristic hormonal abnormalities, primarily manifested as decreased testosterone (T) levels and alterations in gonadotropin levels (**Table 2**).

**Table 2:** Hormonal abnormalities in male psoriasis patients: evidence from existing studies.

Study design	Author, publication year	Sample size	Sex hormones related	Evidence in the literature
Prospective cohort study	Boehncke, 2011 (137)	ncase=23	E2, T, FSH, LH, SHBG	In patients with moderate-to-severe plaque psoriasis, pre-treatment testosterone and SHBG levels were within the normal range, with no evidence of hypogonadism. SHBG was associated with insulin resistance and inflammation, suggesting a metabolic effect rather than a direct disturbance of sex hormone regulation.
Prospective cohort study	Hillary, 2017 (152)	ncase=13	T, SHBG, FSH, LH	A total of 38.5% of patients had total testosterone levels <1 nmol/L; in the obese subgroup (BMI ≥25), 50% had low total testosterone and 37.5% had low free testosterone (<0.25 nmol/L). Low testosterone was associated with obesity and was not specific to psoriasis.
Retrospective cohort study	Liu, 2019 (153)	n=14, 444, ncase=51	T	Prostate cancer patients receiving androgen deprivation therapy (ADT) exhibited a significantly lower risk of psoriasis compared to those not receiving ADT (adjusted HR = 0.52, 95% CI: 0.28–0.95, *p* = 0.035). ADT reduces Th1, Th17 cells, and IL-6 levels, thereby suppressing psoriasis-related inflammation via STAT1, STAT3, and NF-κB signaling pathways.
Retrospective cohort study	Liu, 2018 (154)	n=13, 683, ncase=89	T	Prostate cancer patients receiving androgen deprivation therapy (ADT) did not exhibit an increased risk of psoriasis (adjusted HR = 0.95, 95% CI: 0.63–1.45, *p* = 0.816). ADT may reduce psoriasis occurrence by lowering Th1, Th17 cells, and IL-6 levels, suggesting that a low androgen state may not directly trigger psoriasis.
Case–control study	Allam, 2019 (155)	ncase=121, ncontrol=217	T, SHBG	Among psoriasis patients, 52.1% had low total testosterone levels. Both total and free testosterone levels were significantly lower than those in controls. Testosterone levels were negatively correlated with PASI scores and showed no association with age. Low total testosterone was associated with metabolic syndrome, and patients with PASI ≥10 exhibited significantly lower testosterone levels.
Case–control study	Al-Zubi, 2021 (35)	ncase=88, ncontrol=88	E2, T, FSH, LH	Testosterone levels in the psoriasis group were lower than those in controls (3.8 ± 0.7 ng/mL vs. 5.6 ± 0.9 ng/mL, *P* < 0.001), while estradiol levels were higher (39.7 ± 6.2 pg/mL vs. 25.8 ± 3.7 pg/mL, *P* < 0.001). LH levels were lower in the psoriasis group compared to controls (3.8 ± 1.1 mIU/L vs. 4.1 ± 0.2 mIU/L, *P* = 0.009), whereas FSH levels were higher (5.1 ± 1.6 mIU/L vs. 4.0 ± 1.0 mIU/L, *P* < 0.001). Age and PASI score were significant predictors of sperm concentration (*P* < 0.001). Hormonal abnormalities may be associated with psoriasis-related inflammation or reproductive dysfunction.
Case–control study	Özer, 2020 (156)	ncase=87, ncontrol=99	E2, T	Male patients with psoriasis exhibited significantly higher 2D:4D digit ratios compared to controls (right hand: 0.976 ± 0.025 vs. 0.969 ± 0.023, *P* = 0.009; left hand: 0.969 ± 0.033 vs. 0.961 ± 0.022, *P* = 0.001), suggesting that lower fetal testosterone or higher estrogen exposure may increase the risk of developing psoriasis.
Case–control study	Caldarola, 2017 (34)	ncase=50, ncontrol=50	E2, T, SHBG, FSH, LH	In untreated patients with moderate-to-severe plaque psoriasis, testosterone and SHBG levels were significantly lower than those in controls, while E2 levels were elevated; no significant differences were observed in FSH or LH levels, and no significant correlations were found between sex hormone levels and PASI scores. Ultrasound evidence of accessory gland inflammation was present in 6 of 50 patients, suggesting a potential impact of inflammation on fertility.
Case–control study	Eltaweel, 2018 (32)	ncase=50 ncontrol=30	E2, T, DHT	Total testosterone levels in psoriasis patients were significantly lower than those in controls (316.50 ± 106.88 vs. 793.25 ± 116.70 ng/dL, *P* < 0.001); estradiol levels were significantly higher (*P* < 0.05); and DHT levels were significantly elevated (*P* < 0.001). Total testosterone was positively correlated with age (*P* = 0.042), and estradiol was positively correlated with BMI (*P* = 0.008), but no significant correlation was observed between estradiol and PASI scores (*P* = 0.897). Erectile function was significantly impaired (*P* = 0.046).
Cross-sectional study	Cemil, 2015 (30)	ncase=47, ncontrol=20	E2, T, FSH, LH	In patients with plaque psoriasis, testosterone levels were lower, while E2 levels were significantly higher and negatively correlated with PASI scores. FSH and LH levels were slightly elevated, but the differences were not statistically significant.
Cross-sectional study	Nahidi, 2023 (31)	ncase=30, ncontrol=30	E2, T, FSH, LH	In patients with chronic plaque psoriasis, LH and FSH levels were significantly higher than those in controls, while testosterone and estradiol levels were significantly lower. Testosterone levels correlated with nail and joint involvement as well as PASI scores, whereas LH, FSH, and estradiol showed no significant associations.
Cross-sectional study	Schwarz, 1981 (29)	ncase=33, ncontrol=8	T, DHEA	Serum testosterone levels in patients with psoriasis were slightly lower than those in healthy controls, although the difference was not statistically significant. The reduction in dehydroepiandrosterone (DHEA) levels was more likely attributable to increased 17β-hydroxysteroid dehydrogenase activity rather than decreased DHEA synthesis.
Case	Saad, 2015 (33)	ncase=15	T	In male psoriasis patients with hypogonadism, testosterone replacement therapy resulted in a reduction of at least 75% in PASI scores, and the PGA score decreased from 4.9 ± 0.8 to 0.8 ± 0.3 (*P* < 0.001). Testosterone therapy may exert anti-inflammatory effects and improve symptoms of psoriasis.
Case	Buttars, 2016 (157)	ncase=2	T	Two prostate cancer patients developed psoriasiform eruptions following treatment with enzalutamide and apalutamide, respectively, suggesting that androgen receptor antagonism may trigger psoriasiform skin reactions.

Decreased testosterone levels are the most common hormonal abnormality in male psoriasis patients. Early prospective cohort studies showed that testosterone levels in male patients with severe psoriasis were lower than in healthy controls, although the difference was significant only at specific time points (29). Subsequent studies confirmed this finding, with cross-sectional studies demonstrating significantly lower testosterone levels in male patients with plaque psoriasis compared to controls, inversely correlated with Psoriasis Area and Severity Index (PASI) scores (30, 31). A case-control study further indicated that testosterone levels were significantly reduced in male patients with moderate-to-severe psoriasis and were associated with erectile dysfunction (32). A clinical intervention study revealed that testosterone supplementation in psoriasis patients with hypogonadism improved skin lesions, with PASI scores significantly decreased over 24 months, supporting a protective role of testosterone in psoriasis (33).

Findings regarding estradiol levels in male psoriasis patients are inconsistent. Some studies have reported elevated estradiol levels, inversely correlated with PASI scores, suggesting a possible compensatory conversion mediated by aromatase (30, 32, 34, 35). However, recent cross-sectional studies have shown that estradiol levels are reduced in male patients with moderate-to-severe plaque psoriasis, challenging the universality of the compensatory conversion mechanism (31).

Results concerning gonadotropins FSH and LH are similarly heterogeneous. Some cross-sectional studies have indicated elevated FSH and LH levels in male psoriasis patients, suggesting feedback regulation within the hypothalamic-pituitary-gonadal axis (30, 31). However, other case-control studies have found no significant differences, with some even reporting decreased LH levels (34, 35).

From a biological perspective, the reduced testosterone levels frequently observed in male psoriasis patients may not solely reflect chronic inflammation but also interact with metabolic abnormalities that are common in this population. Low testosterone has been associated with increased visceral adiposity, insulin resistance, and endothelial dysfunction in other inflammatory diseases, suggesting that hypogonadism may both result from and contribute to the broader inflammatory–metabolic milieu of psoriasis (36–38). The inconsistent findings regarding estradiol levels may additionally relate to differences in body composition and variable aromatase activity among patients, implying that local hormone conversion and tissue-specific metabolism could influence circulating hormone levels (39).

From a methodological standpoint, discrepancies across studies may partly arise from variation in sampling protocols and laboratory measurements. Testosterone exhibits a pronounced circadian rhythm, is influenced by acute stress and illness, and differs significantly between total and free fractions, but many studies did not standardize sampling time or account for sex hormone–binding globulin (40, 41). Differences in assay technique, disease severity stratification, and adjustment for factors such as age, body mass index (BMI), smoking, and medications—particularly corticosteroids and systemic agents capable of suppressing the HPA axis—likely further contribute to the heterogeneity of findings. These considerations emphasize the need for harmonized study designs when interpreting hormonal alterations in male psoriasis patients.

**Figure 1:**
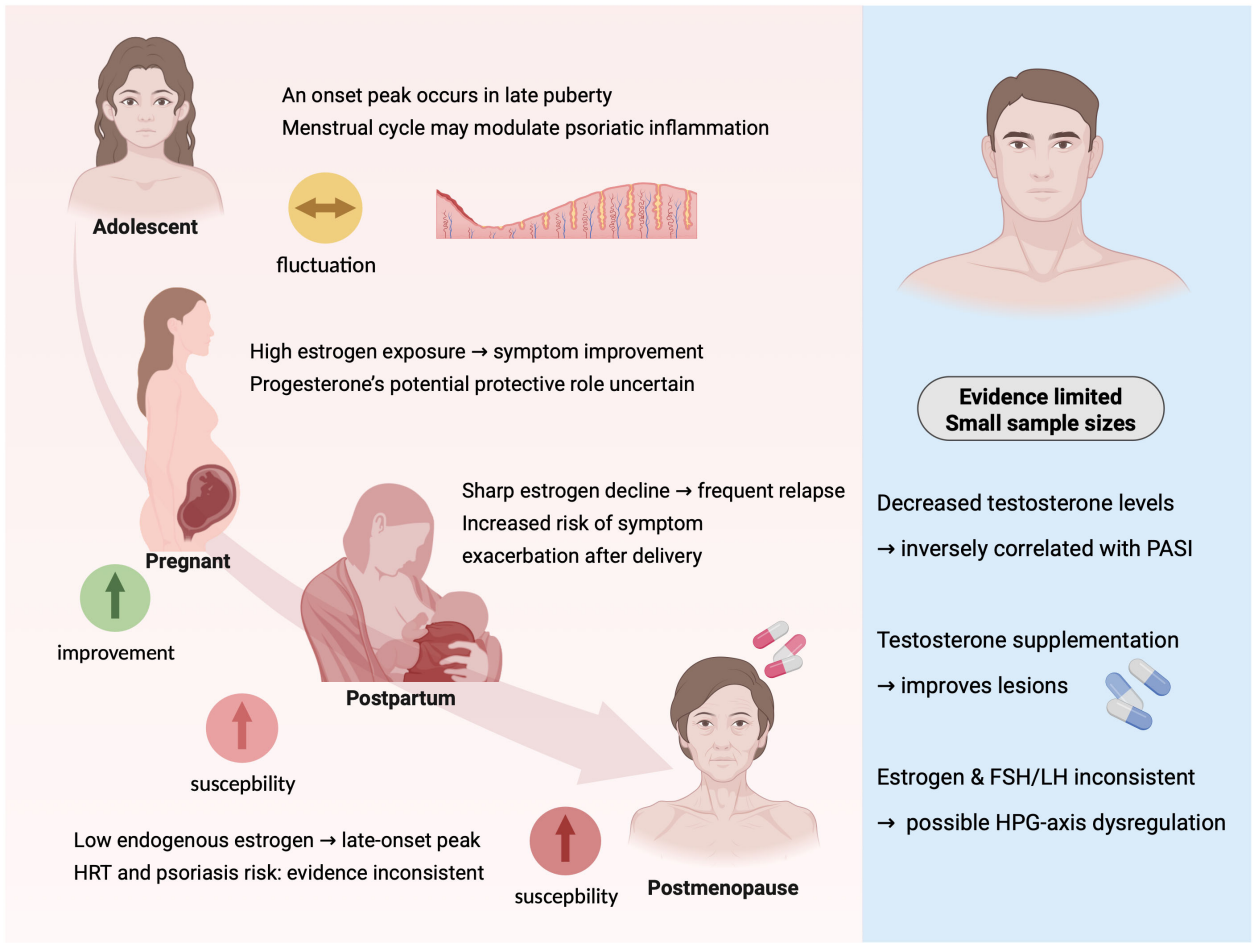
Clinical evidence of sex hormone-associated modulation of psoriasis throughout the life course. This schematic summarizes key clinical patterns linking sex hormone fluctuations to psoriasis onset, progression, and severity across the female and male life spans. In females (left panel), disease onset often peaks in late puberty and manopause, with symptom fluctuation potentially influenced by the menstrual cycle. During pregnancy, elevated estrogen levels are commonly associated with symptom improvement, whereas the postpartum period is characterized by a sharp decline in estrogen and a heightened risk of relapse. In postmenopausal women, decreased endogenous estrogen levels may increase psoriasis susceptibility, though findings regarding hormone replacement therapy (HRT) remain inconsistent. In males (right panel), clinical studies report decreased serum testosterone levels, which are inversely correlated with disease severity (PASI scores), and case studies suggest that testosterone supplementation may improve psoriatic lesions. However, evidence remains limited by small sample sizes and heterogeneity across studies. (Created with BioRender.com).

Together, these clinical observations highlight the critical role of sex hormone dynamics across the lifespan in shaping psoriasis onset and progression. A summary of these hormone–disease associations is provided in **Figure 1**.

## 3 Mechanisms of sex hormone-mediated regulation of immune inflammation in psoriasis

**Figure 2:**
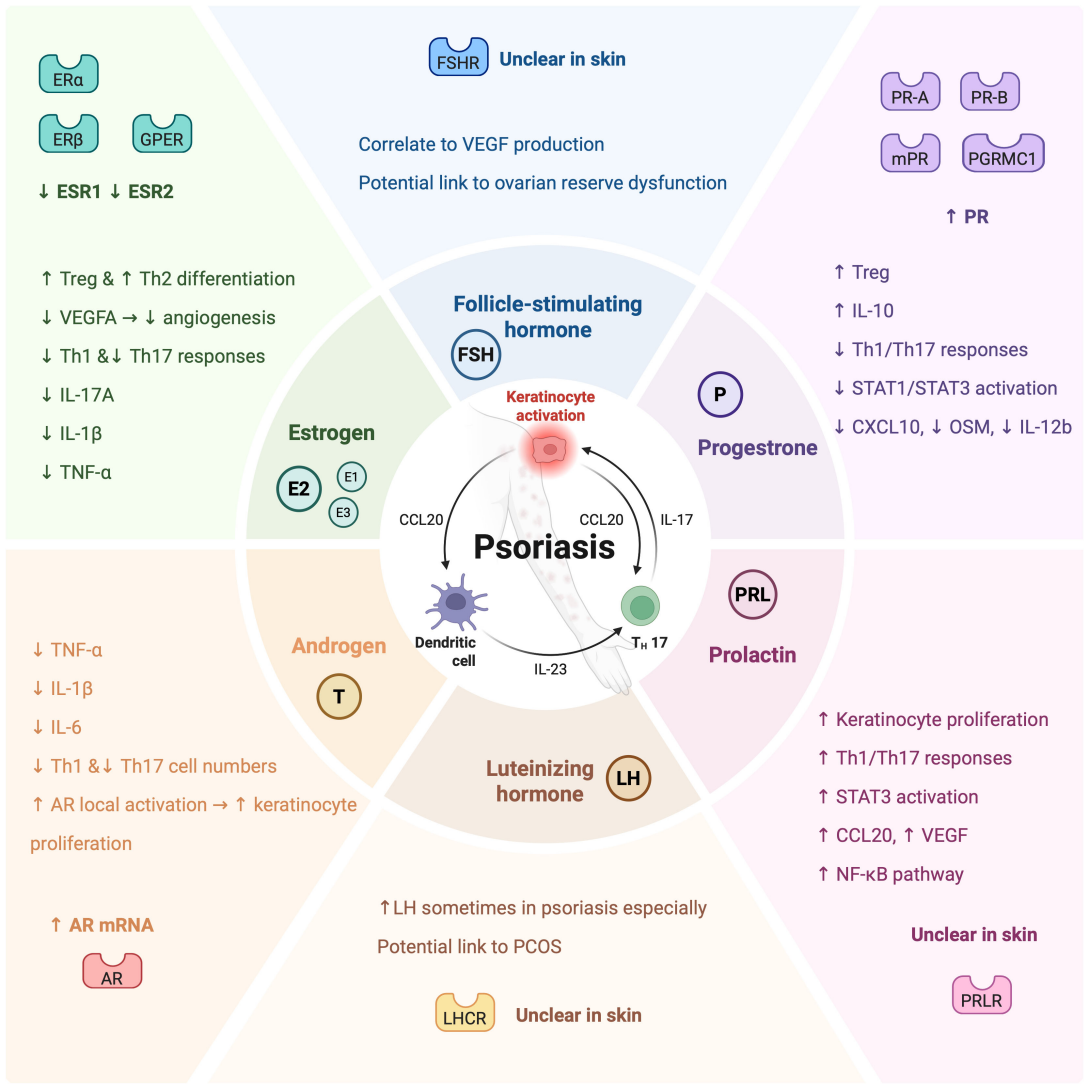
Mechanisms of sex hormone-mediated regulation of immune inflammation in psoriasis. Sex hormones regulate immune and inflammatory pathways implicated in psoriasis pathogenesis through distinct receptor signaling and cellular targets. Estrogen promotes Treg and Th2 cell differentiation, while suppressing Th1 and Th17 responses, reducing the expression of proinflammatory cytokines (IL-17A, IL-1β, TNF-α), and inhibiting VEGFA-mediated angiogenesis. Androgens suppress Th1/Th17-associated cytokines and cell numbers; however, local androgen receptor (AR) activation may paradoxically enhance keratinocyte proliferation. Progesterone exerts anti-inflammatory effects by enhancing Treg differentiation and IL-10 production, while inhibiting Th1/Th17 polarization, STAT1/STAT3 activation, and downstream inflammatory mediators such as CXCL10, OSM, and IL-12b. In contrast, prolactin promotes keratinocyte proliferation, activates STAT3 signaling, and enhances Th1/Th17 responses, CCL20 secretion, and VEGF-mediated angiogenesis, possibly amplifying psoriatic inflammation. Follicle-stimulating hormone (FSH) may induce VEGF production and contribute to angiogenesis, with emerging links to ovarian reserve dysfunction. Luteinizing hormone (LH) is occasionally elevated in psoriasis patients, potentially associated with polycystic ovary syndrome (PCOS) comorbidity. The central schematic illustrates the key interplay between dendritic cells, Th17 cells, and keratinocytes via IL-23, IL-17, and CCL20, forming a feed-forward loop in psoriasis that is modulated by sex hormone signaling. (Created with BioRender.com).

Sex hormones regulate immune inflammation in psoriasis through diverse pathways involving both innate and adaptive immune cells. Their immunomodulatory effects are mediated by receptor-specific signaling, differing across hormone types. An overview of these regulatory mechanisms is illustrated in **Figure 2**. Importantly, these hormone-mediated pathways are influenced by systemic factors, such as systemic inflammatory states, metabolic dysregulation, and stress-related neuroendocrine changes, as discussed in Section 4.

### 3.1 Estrogen-mediated regulation of immune inflammation in psoriasis

Estrogen exerts its effects in psoriasis through multiple cell types and molecular pathways. This section systematically explores the types and distribution of estrogen receptors (Estrogen Receptor 1, ESR1; Estrogen Receptor 2, ESR2), the effects of estrogen on immune cells and keratinocytes, and the specific roles of ESR1 and ESR2 in psoriasis, aiming to elucidate their regulatory mechanisms in the pathogenesis of psoriasis.

Estrogen exists in three primary physiological forms: estrone (E1), 17β-estradiol (E2), and estriol (E3), with E2 being the most abundant and bioactive during reproductive age (42). Estrogen receptors include nuclear receptors ERα and ERβ, as well as the membrane-bound G protein-coupled estrogen receptor (GPER or GPR30), which are widely distributed in tissues throughout the body, including keratinocytes, dendritic cells, and monocytes/macrophages in the skin (12, 43). The effects of estrogen vary depending on cell type, receptor expression, and hormone levels, and its relationship with skin physiology has been widely investigated, including its roles in enhancing skin barrier function and promoting wound healing (44). In the context of psoriasis, estradiol has been found to promote the differentiation and function of regulatory T cells (Tregs) and inhibit the secretion of inflammatory cytokines by neutrophils and macrophages.

Psoriasis is driven by inflammation mediated by Th1 and Th17 cells, and estrogen plays a significant role in psoriasis by modulating immune cell function (45). High estrogen levels during pregnancy are associated with improvement in various Th1- and Th17-mediated immune diseases, a trend that has been observed in human populations (46). E2 induces polarization of CD4+ T cells toward Th2 and Tregs, upregulating the expression of Th2-related genes (e.g., GATA3, IL-4) and Treg-related genes (e.g., Foxp3, IL-10), while suppressing Th1-related genes (e.g., T-bet, IFN-γ) and Th17-related genes (e.g., ROR-γt, IL-17). This process has been observed in pre-clinical studies, including animal models and *in vitro* experiments, which provide evidence of E2’s role in immune modulation (43, 47). Additionally, Adachi et al (48) found that E2, by acting on estrogen receptors (ER) in neutrophils and macrophages, inhibits IL-1β production by these cells, thereby reducing IL-17A production and alleviating psoriasis-like inflammation in mice models. Estrogen also regulates the development and function of the cDC2 subset through the ERα signaling pathway, potentially suppressing its overactivation, which reduces the production of proinflammatory cytokines and T cell activation, mitigating the inflammatory response in psoriasis, as demonstrated in animal models (49). Beyond its effects on cytokine production, E2 has also been reported to inhibit neutrophil functions, such as superoxide anion generation, degranulation, and migration, as shown in *in vitro* studies (50).

The histological features of psoriatic lesions include excessive keratinocyte proliferation and increased dermal vasculature (4). The effects of E2 on keratinocytes are dual, promoting proliferation while potentially mitigating excessive proliferation indirectly through anti-inflammatory actions (51). In a mannan-induced female psoriasis mouse model, E2 exacerbates psoriasis-like inflammation by upregulating ERβ and proinflammatory miRNAs (e.g., miR-21), thereby promoting keratinocyte proliferation (52). However, *in vitro* studies show that genistein, an isoflavone compound with estrogen-like effects, reduces the expression of CCL20, S100A7, and S100A9 induced by IL-17A and TNF-α by inhibiting the NF-κB pathway, exhibiting anti-inflammatory and anti-proliferative effects, and demonstrating therapeutic potential in patients with mild to moderate psoriasis (53–55). Angiogenesis in psoriatic lesions is a key early pathological feature, with vascular endothelial growth factor (VEGF) playing a critical role (56). 2-Methoxyestradiol, an endogenous estrogen metabolite, reduces VEGFA expression by inhibiting VEGFR1 and VEGFR2, thereby suppressing angiogenesis and demonstrating anti-psoriatic potential, as shown in animal and *in vitro* studies (57, 58).

ERα and ERβ are encoded by the ESR1 and ESR2 genes, respectively. Transcriptomic analysis reveals that ESR1 and ESR2 expression is significantly downregulated in both lesional and non-lesional skin of psoriasis patients (59). Downregulation of ESR1 may play a critical role in psoriasis pathogenesis, and its restored expression is closely associated with symptom amelioration and relapse prevention, suggesting ESR1 as a potential therapeutic target (60). However, some studies have found that inhibiting ESR1 expression can alleviate psoriasis symptoms, a contradiction that may stem from differences in study models, ESR1 isoform specificity, or complex interactions with inflammatory signaling pathways (61). Future research is needed to further elucidate the precise mechanisms of ESR1 in psoriasis. Changes in ESR2 expression in psoriatic skin are less pronounced than those of ESR1, but its interactions with transcription factors such as PPARA and SREBF may indirectly influence disease progression by regulating lipid metabolism (62). In the context of common comorbidities like metabolic syndrome, which is prevalent in psoriasis patients, systemic factors such as obesity can impair estrogen bioavailability through enhanced aromatase conversion in adipose tissue, potentially diminishing its protective anti-inflammatory effects on immune cells (63). Similarly, associated depressive states may exacerbate this via stress-induced HPA axis interactions, altering receptor expression and signaling efficiency (64). Whether the association of ESR2 with lipid metabolism offers new perspectives on psoriasis comorbidities, such as cardiovascular disease, remains to be explored.

### 3.2 Regulatory roles of androgen signaling in psoriasis

Androgens, including testosterone and its active metabolite dihydrotestosterone (DHT), play a significant role in skin physiological functions, with their involvement in the pathogenesis of psoriasis being complex and controversial. The androgen receptor (AR), a member of the nuclear receptor superfamily, is widely expressed in keratinocytes, sebocytes, dermal fibroblasts, and immune cells. Upon ligand binding, AR forms homodimers, translocates to the nucleus, and regulates the expression of genes involved in sebaceous gland function, hair follicle development, epidermal differentiation, and inflammatory responses (65). Unlike estrogen receptors, which have been extensively characterized in immune regulation, the precise roles of AR in psoriatic inflammation remain incompletely understood, with most evidence derived from studies in other inflammatory or autoimmune conditions.

Systemic androgen signaling is predominantly anti-inflammatory. Clinical studies have reported lower serum testosterone levels in male psoriasis patients, correlating with greater disease severity (30, 31). In immune cells, AR signaling suppresses macrophage production of TNF-α, IL-1β, and IL-6, promotes M2-like polarization, and attenuates dendritic-cell maturation, thereby limiting Th1/Th17 activation, as demonstrated in pre-clinical studies (66–68). In CD4^+^ T cells, AR activation inhibits Th1 and Th17 differentiation while stabilizing regulatory T cells via transcriptional modulation of IFN-γ, IL-17A, RORγt, and Foxp3 (69, 70). Together, these findings support a model in which systemic androgen deficiency may predispose male patients to enhanced inflammatory responses and greater psoriasis disease burden. However, the clinical evidence for testosterone therapy in psoriasis remains limited and inconclusive.

In contrast to its systemic immunosuppressive actions, AR signaling within the skin may exert pro-inflammatory effects that are particularly relevant to psoriatic lesion formation. Human transcriptomic analysis reveals that AR mRNA expression levels are significantly higher in psoriatic lesions compared to non-lesional skin and healthy skin, and the balance between androgens and estrogens is shifted toward androgens, potentially associated with the chronic inflammatory state of the disease (59). AR activation in keratinocytes has been reported to interact with MAPK and NF-κB signaling, modulating the expression of cytokines and chemokines such as IL-8 and CXCL family members that contribute to neutrophil recruitment and epidermal hyperplasia—hallmark features of psoriatic lesions (71). Disturbances in AR-mediated calcium signaling have also been proposed to drive excessive keratinocyte proliferation. Ligand-independent AR activation may also occur in inflamed skin, as insulin-like growth factor-1 (IGF-1) can activate AR via phosphorylation cascades (72, 73). Although these mechanisms require direct validation in psoriasis models, they provide a plausible framework for understanding how local AR signaling may contribute to lesion development.

Clinical observations regarding androgen manipulation appear contradictory, with AR antagonists occasionally triggering psoriasis flares while androgen deprivation therapy (ADT) has been associated with reduced disease risk. Rather than reflecting true inconsistency, these patterns may indicate divergent roles of systemic androgen deficiency versus local AR activity within the skin and immune system. This distinction supports the concept that systemic androgens exert predominantly anti-inflammatory effects, whereas enhanced local AR activation in psoriatic skin may promote keratinocyte-driven inflammation. In this context, metabolic syndrome and obesity—frequent in psoriasis—can disrupt androgen function by inducing insulin resistance, thereby lowering testosterone levels and amplifying local pro-inflammatory AR signaling (74, 75). This effect may be further compounded by depressive states, which indirectly suppress androgen production through chronic stress (21).

Taken together, current evidence supports a multidimensional model in which androgens exert systemic anti-inflammatory effects through immune modulation, whereas increased local AR expression and activation in psoriatic skin may enhance keratinocyte-driven inflammation. This dualistic framework may help reconcile clinical paradoxes and underscores the importance of examining androgen signaling across both systemic and cutaneous contexts when evaluating its role in psoriasis pathogenesis.

### 3.3 Immunomodulatory effects of progesterone in psoriasis

The biological effects of progesterone (P4) are mediated through multiple receptor systems. Classical progesterone receptors (PR), including the nuclear receptor subtypes PR-A and PR-B, are expressed in keratinocytes, fibroblasts, and other cells, regulating gene expression by binding to progesterone response elements (PRE) in gene promoters, which involves processes such as cell proliferation, differentiation, and apoptosis. Membrane progesterone receptors (mPR), comprising five subtypes, belong to the seven-transmembrane G protein-coupled receptor family and mediate rapid non-genomic effects. Additionally, auxiliary receptors such as progesterone receptor membrane component-1 (PGRMC1) also participate in signal transduction. These receptors exhibit specific expression patterns in different cells and tissues, and changes in their relative expression ratios may influence the intensity and directionality of P4’s biological effects (76).

P4 exerts immunomodulatory effects by regulating T-cell subset differentiation and cytokine networks (77). P4 tends to shift CD4+ T-helper cell responses from a Th1 to a Th2 phenotype and promotes the production of anti-inflammatory cytokines IL-4 and IL-10. Treatment of umbilical cord blood cells with P4 increases the proportion of FOXP3+ Treg cells, thereby enhancing immune tolerance, while reducing the number of pro-inflammatory Th17 cells (78). Hellberg et al (79) demonstrated through *in vitro* experiments that P4 significantly inhibits CD4+ T-cell activation and downregulates the expression of STAT1 and STAT3. These transcription factors play critical roles in the pathogenesis of psoriasis, and the expression of their target genes is also modulated by P4. Additionally, P4 significantly downregulates the expression of inflammation-related genes and proteins, such as IL-12b, CXCL10, and OSM, thereby suppressing inflammatory responses.

In psoriatic lesional skin, the expression levels of PR are altered, with an upregulation of PR expression particularly observed in suprabasal keratinocytes of the epidermis (80). However, the precise role of PR in the pathogenesis of psoriasis and its activation mechanisms remain to be further elucidated. PR gene polymorphisms affect receptor protein stability and transcriptional activity, representing a key factor in inter-individual differences in progesterone sensitivity (81). This may explain the variability in symptom improvement among psoriasis patients during pregnancy. Such variability could be exacerbated by systemic comorbidities like metabolic syndrome, where obesity alters progesterone metabolism via hepatic enzyme changes, potentially diminishing its immunomodulatory benefits (82, 83). Additionally, depressive psychological states linked to psoriasis may influence this through neuroendocrine interactions (64). However, systematic studies in psoriasis patient populations are currently lacking, which could be an important direction for future research into personalized treatment strategies.

### 3.4 Potential role of prolactin in psoriasis

Prolactin (PRL) is a polypeptide hormone primarily secreted by the anterior pituitary, traditionally associated with mammary gland development and lactation. However, recent studies indicate that PRL exerts immunostimulatory effects, potentially contributing to the pathogenesis of various autoimmune diseases (84). PRL exists in three main forms: monomeric PRL (~23 kDa), big PRL (50–60 kDa), and macro-PRL (>100 kDa). Beyond the pituitary, PRL can also be synthesized in peripheral tissues and immune cells. The prolactin receptor (PRLR), a member of the type I cytokine receptor family, is widely distributed in immune system cells and skin tissues. The binding of PRL to PRLR activates signaling pathways such as JAK2/STAT5, MAPK, and PI3K/AKT, regulating cell proliferation, differentiation, and inflammatory responses (84).

Multiple studies suggest that serum PRL levels in psoriasis patients may be associated with disease severity, duration, and treatment response, a trend observed in clinical studies (85). A 2018 meta-analysis, including 12 case-control studies, found significantly elevated circulating PRL levels in psoriasis patients (SMD = 0.54), with a positive correlation to PASI scores, though without statistical significance, indicating mixed evidence from clinical data (86). Additionally, some psoriasis patients with concurrent prolactinomas showed marked improvement in skin lesions following bromocriptine treatment, suggesting that PRL may play a role in regulating disease activity (87). However, existing clinical studies exhibit heterogeneity in sample size, sex stratification, and patient subtypes, leading to controversies in result interpretation. Future multicenter, prospective studies are needed to further elucidate the associations between different PRL isoforms, psoriasis phenotypes, and treatment responses.

Mechanistic studies suggest that prolactin may contribute to the pathogenesis and progression of psoriasis by stimulating keratinocyte proliferation, enhancing Th1 and Th17 responses, and promoting VEGF production, findings supported by experimental models, including both *in vitro* and animal studies (86). Local skin mRNA and protein expression of PRL are significantly elevated in psoriatic lesions, with no significant correlation to serum PRL levels, indicating that locally synthesized PRL may exert an independent role (88, 89). *In vitro* experiments demonstrate that PRL stimulates proliferation of cultured keratinocytes, and in the late stages of keratinocyte differentiation, PRLR expression is upregulated, further amplifying PRL’s biological effects (90). Animal studies further support the pathogenic role of PRL in psoriasis. In imiquimod-induced psoriasis mouse models, exogenous PRL promotes STAT3 activation, enhances Th17 cell differentiation, and significantly upregulates the expression of IL-17A, IL-22, and CCL20 (91). Additionally, studies indicate that PRL synergizes with IL-17 to stimulate keratinocytes to release CCL20 by enhancing NF-κB pathway activity, thereby recruiting more Th17 cells and forming a positive inflammatory feedback loop (92). Furthermore, PRL influences angiogenesis, with 23 kDa PRL promoting angiogenesis, while its cleavage product, 16 kDa PRL, exhibits anti-angiogenic effects by suppressing VEGF expression and inducing endothelial cell apoptosis (90).

Within systemic contexts, metabolic syndrome can heighten PRL’s pro-inflammatory role through elevated adipokines like leptin, which correlates with psoriasis severity, a trend supported by clinical studies (93, 94). Depressive states may amplify this by increasing PRL release via stress pathways, perpetuating immune dysregulation (95). However, the receptor distribution, local synthesis sources, and dynamic regulatory mechanisms of PRL in different skin cell types remain unclear. Animal studies have primarily focused on exogenous PRL interventions, lacking tissue-specific PRLR knockout models. Future research should employ genetic manipulation, single-cell sequencing, and spatial transcriptomics to further elucidate the specific roles of PRL/PRLR signaling in the skin inflammatory microenvironment (96). Notably, PRL is also a neuroendocrine factor, with its synthesis and secretion regulated by the hypothalamic-pituitary axis (97). Under stress conditions, activation of the brain-skin axis can increase PRL release, potentially influencing the onset and exacerbation of psoriasis, providing a new research perspective on the association between psoriasis and psychological factors. This field currently lacks experimental validation and clinical longitudinal observations. Future studies could integrate psychological stress models, neuroendocrine regulatory pathways, and behavioral interventions to further explore the mechanistic role of PRL in stress susceptibility in psoriasis (98).

### 3.5 Possible roles of FSH and LH in psoriasis

FSH and LH are glycoprotein hormones essential for human reproduction, primarily activating G protein-coupled receptors FSHR and LHCGR. These hormones regulate cell metabolism and steroid hormone production through classical signaling pathways (Gs/cAMP/PKA) and potentially through non-canonical pathways such as PI3K/AKT (99, 100). While both FSHR and LHCGR are predominantly expressed in gonadal tissues, recent studies have drawn attention to the potential expression of these receptors in extragonadal tissues, including skin; however, their roles in psoriatic skin remain unclear (101–103).

Hypothetical mechanisms have suggested that FSH and LH may influence psoriasis pathophysiology through their effects on immune cells and keratinocyte function. Activation of the Gs/cAMP/PKA axis could modulate immune responses, potentially impacting dendritic cells or Th17-associated cytokines involved in psoriasis (104). Additionally, FSH has been shown to activate the PI3K/AKT pathway in certain contexts, which could contribute to keratinocyte hyperproliferation, a key feature of psoriatic lesions (105, 106). Elevated FSH has also been associated with increased VEGF production, implicating gonadotropin signaling in psoriatic angiogenesis (30, 31).

While the potential for FSH/LH involvement in psoriasis is intriguing, the evidence remains largely speculative. Most studies in this area are limited to small, cross-sectional studies with no direct evidence for FSH/LH receptor expression in psoriatic skin. Notably, research on the relationship between FSH, LH, and psoriasis is still in its infancy, with only preliminary evidence suggesting that elevated gonadotropins may contribute to psoriasis through metabolic and inflammatory pathways, particularly in conditions like polycystic ovary syndrome (PCOS) (107, 108). PCOS is characterized by metabolic disturbances such as insulin resistance, central adiposity, dyslipidemia, and chronic low-grade inflammation, which overlap with features commonly observed in psoriasis (109, 110). These metabolic disturbances could potentially exacerbate psoriasis through interactions with inflammatory pathways, including adipokines and cytokines like leptin, TNF-α, and IL-6, which are known to influence Th17-skewed immunity, a key driver of psoriatic inflammation (111–113). However, these associations are based on indirect findings, and further research is needed to validate these links and clarify the role of gonadotropins in psoriasis.

Given the speculative nature of current evidence, we recommend that the role of FSH and LH in psoriasis be treated with caution and potentially explored in future research. Investigating FSH/LH receptor expression in psoriatic skin, conducting large-scale cohort studies, and exploring the mechanistic pathways *in vitro* and in animal models are crucial next steps to better understand the involvement of gonadotropins in psoriasis pathogenesis.

## 4 Impact of psoriatic inflammation on hormonal homeostasis

### 4.1 Endocrine effects of psoriasis-associated inflammatory cytokines

The persistent inflammatory state of psoriasis is thought to disrupt the biosynthesis and regulation of sex hormones through multiple mechanisms, which may modulate the immunoregulatory pathways outlined in Section 3, such as altering hormone-receptor interactions in immune cells amid systemic comorbidities. However, it is important to note that much of the evidence in this area remains correlational rather than demonstrating direct causality. Clinical observations indicate that reduced serum testosterone levels in male psoriasis patients correlate positively with disease severity, while disease severity in female patients fluctuates with reproductive stages, suggesting a close association between sex hormone status and the inflammatory burden of psoriasis (21, 35). However, it should be noted that these relationships are correlational, and further research is required to determine whether hormonal alterations are direct consequences of psoriasis or secondary to comorbid conditions such as obesity or metabolic syndrome. Basic research further reveals that characteristic pro-inflammatory cytokines of psoriasis, such as IL-17A, IL-23, and IL-12, affect the function of the HPG axis through direct and indirect pathways, leading to significant alterations in sex hormone levels (15).

Animal studies demonstrate that TNF-α injection significantly suppresses the mRNA and protein expression of steroidogenic acute regulatory protein (StAR) in Leydig cells of adult rats, potentially inhibiting testosterone synthesis by blocking cholesterol transport to the inner mitochondrial membrane and inhibiting testosterone synthesis at its source (114). *In vitro* studies further elucidate molecular mechanisms at the cellular level: IL-1β, IL-6, and TNF-α significantly suppress gonadotropin- or cAMP-induced testosterone production in the mouse Leydig cell line TM3. This is accompanied by downregulated expression of key steroidogenic enzymes, including StAR, P450scc, P450c17, and 3β-HSD, in a dose-dependent manner (115). Additionally, the inhibitory effect of inflammatory cytokines on testosterone synthesis is associated with elevated LH levels, suggesting that inflammation disrupts the feedback regulation of the HPG axis, simultaneously suppressing peripheral sex hormone synthesis and activating central compensatory responses (116).

In fact, a widespread pathophysiological link exists between inflammatory states and sex hormone dysregulation. Data from the National Health and Nutrition Examination Survey population show that, in males, inflammatory indices such as the systemic immune-inflammation index, neutrophil-to-lymphocyte ratio, and the product of platelet count and neutrophil count are negatively correlated with testosterone levels. However, this cross-sectional study does not imply causality, and it remains uncertain whether the inflammatory indices are directly contributing to reduced testosterone levels or merely reflecting underlying processes. In females, inflammatory indices are negatively associated with sex hormone-binding globulin (SHBG) and testosterone, suggesting that inflammation impacts the bioavailability of sex hormones (117). Notably, studies on similar immune-inflammatory diseases indicate that the female HPG axis appears more resilient to immune-inflammatory perturbations than the male counterpart. This sex difference may be related to the anti-inflammatory properties of estrogens and female-specific hormonal feedback regulation mechanisms (116). For systemic inflammatory diseases, including psoriasis, inflammatory indices may serve as effective predictors of sex hormone dysregulation, providing valuable references for clinical assessment and intervention.

### 4.2 Interaction between stress response and the HPA–HPG axis in psoriasis

The concept of the brain-skin axis highlights the bidirectional interplay between the central nervous system, immune system, and skin, where psychological stress acts as a pivotal modulator in inflammatory dermatoses like psoriasis (118). This axis involves neuroendocrine pathways, including the release of neuropeptides such as substance P and nerve growth factor from sensory nerves in the skin. These peptides amplify local inflammation and interact with systemic stress responses via the HPA axis (95). In psoriasis, chronic stress exacerbates disease flares by promoting a pro-inflammatory milieu in the skin, while psoriatic inflammation can feedback to the brain, heightening perceived stress and perpetuating a vicious cycle (118). Psychological stress, a significant trigger and exacerbating factor for psoriasis, further disrupts patients’ sex hormone balance through complex interactions between the HPA and HPG axes. Psoriasis patients exhibit evident HPA axis dysfunction. Clinical studies show that, under high-stress conditions, psoriasis patients display lower cortisol levels. The adrenocorticotropic hormone (ACTH)/cortisol ratio is positively correlated with PASI scores, indicating that the degree of impaired HPA axis responsiveness is associated with disease severity (119). Studies on acute social stress reveal that stress-sensitive psoriasis patients have lower baseline salivary cortisol levels and reduced post-stress serum cortisol levels, a change that may predispose patients to psoriasis relapse (120).

The HPA and HPG axes exhibit extensive mutual regulation at the hypothalamic and pituitary levels. Under chronic stress, persistently elevated cortisol suppresses reproductive axis function through multiple mechanisms. First, glucocorticoids inhibit the pulsatile secretion of gonadotropin-releasing hormone (GnRH) at the hypothalamic level. Second, they reduce gonadotroph responsiveness to GnRH and downregulate GnRH receptor expression at the pituitary level, decreasing LH and FSH secretion (14). Furthermore, chronic inflammation in psoriasis intersects with this suppression. Pro-inflammatory cytokines like IL-6 and TNF-α, elevated in psoriatic patients, can directly impair hypothalamic GnRH neurons and promote glucocorticoid resistance in gonadal tissues, amplifying HPG axis inhibition (95, 121). Stress-induced glucocorticoids also upregulate gonadotropin inhibitory hormone expression in the hypothalamus, providing an additional pathway for reproductive suppression during prolonged inflammatory states (122). Animal studies indicate that glucocorticoids also directly affect gonadal tissues. In females, cortisol acts on ovarian granulosa cells, inhibiting aromatase activity, reducing E2 synthesis, and impairing follicular development. In males, glucocorticoids directly suppress steroidogenesis in testicular Leydig cells, leading to decreased testosterone levels (123). Additionally, sex hormones reciprocally modulate HPA axis function. Androgens reduce HPA axis activity by inhibiting the synthesis of corticotropin-releasing hormone (CRH) and ACTH (124). Conversely, estrogens may enhance HPA axis stress responsiveness by increasing CRH gene expression (125). These mechanisms may result in higher cortisol levels and stronger HPA axis responses in females under chronic stress, exacerbating psoriasis, particularly during reproductive stage fluctuations. In contrast, androgens may confer a protective effect in males by suppressing HPA axis activity. These sex differences not only explain variations in the clinical presentation of psoriasis between sexes but also provide potential directions for developing sex-specific treatment strategies.

However, current research on the specific mechanisms of HPA-HPG axis interactions in psoriasis remains relatively limited. Future studies should focus on the reciprocal regulation of the HPA axis by sex hormone changes in psoriasis patients The restorative effects of anti-psoriatic treatments (such as biologics) on the neuroendocrine axis, and how specific inflammatory pathways, such as Th17 cells, influence disease progression through the HPA-HPG axis, should also be explored. Viewing sex hormone profiles as systemic biomarkers of psoriasis severity could enhance clinical monitoring, as disruptions in the brain-skin axis and HPA-HPG crosstalk correlate with disease flares and therapeutic outcomes (126). These efforts will provide a more comprehensive perspective on the complex relationship between psoriasis and sex hormones.

It is also critical to distinguish disease-intrinsic neuroendocrine dysregulation from iatrogenic effects; a confounding factor frequently encountered in clinical studies. Glucocorticoids, widely used in topical and systemic psoriasis management, exert potent negative feedback on the hypothalamus and pituitary, suppressing endogenous ACTH and cortisol secretion (127). This iatrogenic HPA axis suppression can subsequently inhibit the HPG axis, leading to hypogonadotropic hypogonadism manifested by reduced LH, FSH, and testosterone levels (128). Consequently, the “low cortisol” or “low testosterone” profiles observed in some severe psoriasis cohorts may reflect the cumulative impact of prior corticosteroid therapy rather than, or in addition to, stress-induced suppression. Ignoring this treatment-related confounder may lead to misinterpretation of hormonal baselines, underscoring the need for careful stratification by treatment history in future research.

### 4.3 Metabolic dysregulation as a mediator of hormonal imbalance in psoriasis

Psoriasis is closely associated with metabolic syndrome, which encompasses obesity, dyslipidemia, hypertension, and impaired glucose tolerance. Studies indicate that the prevalence of metabolic syndrome in psoriasis patients is as high as 32% and correlates positively with disease severity (129). Metabolic dysregulation, including insulin resistance and adipose tissue dysfunction, mediates sex hormone imbalances through multiple mechanisms, interacting with inflammatory pathways discussed in Sections 4.1 (inflammatory factors) and 4.2 (stress response).

In psoriasis, the persistent systemic inflammation is not only a consequence of immune dysregulation but also exacerbates metabolic dysfunction, influencing sex hormone levels in a manner that overlaps with conditions like PCOS (107). PCOS is characterized by insulin resistance, central obesity, and dyslipidemia—common features also observed in psoriasis—which can create a synergistic effect in women with both conditions. Elevated pro-inflammatory cytokines and adipokines like leptin contribute to alterations in the HPG axis, which may exacerbate the hormonal dysregulation in women with psoriasis. Notably, this metabolic–inflammatory overlap is particularly relevant to the regulation of estradiol, testosterone, and SHBG levels, and may further modulate the clinical course of psoriasis (51, 130). Intracrine signaling in local tissues plays a critical role here; for instance, aromatase activity in psoriatic skin and adipose tissue is often diminished, leading to reduced conversion of androgens to estrogens and subsequent local estrogen deficiencies that perpetuate inflammation (131). In adipose tissue, hypertrophic adipocytes in obese psoriatic patients exhibit altered aromatase expression, contributing to dysregulated peripheral estrogen synthesis and amplifying systemic hormonal imbalances (132). The co-occurrence of psoriasis and PCOS in women may be linked to a more complex and severe clinical presentation, where the bidirectional effects between metabolic and hormonal dysregulation potentially amplify both conditions. However, this relationship requires further investigation to fully understand its clinical significance.

The interaction between sex hormones and adipose tissue is bidirectional: sex hormones regulate adipose tissue distribution and function, while adipose tissue influences sex hormone metabolism (133). In obese women, elevated insulin levels stimulate ovarian androgen production. Peripheral adipose tissue converts androgens to estrogens via aromatase, negatively regulating the HPO axis and leading to menstrual irregularities and ovulatory dysfunction (134). In psoriasis, metabolic dysregulation and sex hormone imbalances may mutually reinforce each other. For instance, low testosterone levels in male psoriasis patients are associated with elevated leptin and reduced adiponectin levels, while testosterone therapy simultaneously improves inflammatory status and certain adipose factor abnormalities (135, 136). The potential mediating role of metabolic factors in psoriasis and sex hormone dysregulation suggests that interventions targeting metabolic syndrome may concurrently improve disease activity and endocrine disturbances. However, clinical evidence supporting this approach is still limited, and further research is necessary before recommending it as a treatment strategy.

**Figure 3:**
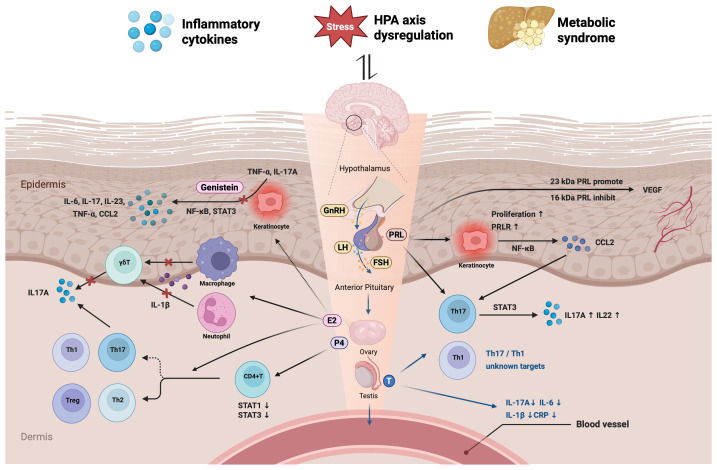
Mechanisms of sex hormone–inflammatory interactions in psoriasis. This schematic illustrates the bidirectional crosstalk between sex hormones and inflammatory pathways in psoriasis. Estradiol (E2), progesterone (P4), testosterone (T), and prolactin (PRL), regulated by the hypothalamic-pituitary-gonadal (HPG) axis, modulate immune responses and keratinocyte behavior. E2 promotes Treg and Th2 differentiation while suppressing Th1/Th17 responses via inhibition of IL-1β and IL-17A. Genistein, a plant-derived estrogenic compound, inhibits NF-κB/STAT3 signaling in keratinocytes, reducing TNF-α, IL-6, and IL-17A. P4 downregulates STAT1/3 activation in CD4+ T cells. T suppresses systemic cytokines (IL-6, IL-1β, CRP), though its cellular targets remain unclear. PRL enhances Th17/Th1 responses and keratinocyte proliferation via NF-κB and STAT3, increasing IL-17A, IL-22, and CCL2. Its isoforms differentially regulate angiogenesis: 23 kDa PRL promotes, while 16 kDa PRL inhibits VEGF. Inflammatory cytokines and stress disrupt the HPG axis, impairing steroidogenesis and hormonal feedback. Metabolic dysregulation further amplifies inflammation and hormone imbalance in psoriasis. (Created with BioRender.com).

Taken together, these findings underscore a complex bidirectional relationship between systemic inflammation, hormonal regulation, stress response, and metabolic dysfunction in psoriasis. This integrative feedback network is schematically represented in **Figure 3**. While sex hormone levels may hold potential as systemic biomarkers for assessing psoriasis severity, this idea remains exploratory and requires further validation before being incorporated into clinical practice. Non-invasive endocrine profiling could, in theory, provide insights into disease progression and guide personalized therapies. However, its clinical application should be approached with caution, and further research is essential to establish its reliability and predictive value (137, 138).

## 5 Conclusions and future directions

This review summarizes the bidirectional interactions between sex hormones and psoriasis, integrating clinical observations with mechanistic insights into hormonal regulation of immune and inflammatory pathways. Disturbances in circulating sex hormones—such as reduced testosterone in men or life-stage–related fluctuations in estrogen and progesterone in women—may influence disease activity, while the chronic inflammatory environment of psoriasis can in turn disrupt normal HPG axis function. However, the extent and nature of these interactions remain exploratory, and further research is needed to confirm their clinical relevance. A clearer understanding of these reciprocal influences may improve our ability to interpret sex-specific clinical patterns and disease variability.

Beyond their pathogenic implications, hormonal pathways may hold relevance for clinical management. Biologic therapies that suppress IL-17 or IL-23 signaling may in principle alleviate cytokine-mediated inhibition of gonadal steroidogenesis, thereby improving hormonal balance in some patients (139). However, this idea remains speculative and requires further validation. Conversely, pre-existing endocrine dysfunction—such as hypogonadism in men or hormonally dynamic reproductive periods in women—may contribute to variability in treatment responses or systemic inflammation profiles (9, 140). Real-world studies further suggest sex-related differences in the use, tolerability, and persistence of systemic and biologic therapies, with men more frequently receiving systemic treatment for more severe disease, whereas women may report higher rates of adverse events and treatment discontinuation (141–144). These considerations highlight the importance of integrating hormonal status into individualized assessment and multidisciplinary management.

Hormone-based therapeutic strategies have shown promise in selected subgroups, including testosterone supplementation in hypogonadal men and estrogen-based interventions in women with cyclical flares (19, 33, 145). However, the long-term efficacy and safety of these interventions remain uncertain, and their application should be considered cautiously. Systemic hormone supplementation carries potential risks, such as thromboembolism and cardiovascular events, which require careful evaluation in psoriasis patients with elevated baseline cardiometabolic risk (25–27, 146). Topical hormone-related interventions may offer safer alternatives, but require further mechanistic and clinical validation.

Several limitations of the current evidence should be acknowledged. Hormone measurements across studies vary widely in assay methodology and sampling timing. They do not consistently account for diurnal rhythm, menstrual cycle phase, or sex hormone–binding globulin levels, or prior exposure to corticosteroids which may suppress baseline HPA/HPG function. These limitations should be carefully considered when interpreting findings and their implications for clinical practice. Most mechanistic findings are extrapolated from non-cutaneous or non-psoriatic models, and large-scale longitudinal datasets capturing dynamic hormone–immune interactions are lacking. These limitations warrant caution when interpreting cross-study comparisons and underscore the need for standardized endocrine assessment frameworks.

Future research should prioritize integrative approaches that unify hormonal, immunologic, neuronal, and metabolic perspectives. Multi-omics, single-cell, and spatial transcriptomic platforms may help delineate cell-type-specific hormone receptor signaling, and clarify synergistic or antagonistic interactions among different hormonal axes. However, it is important to note that the role of sex hormones as systemic biomarkers for psoriasis severity remains an open question and requires further validation. This includes investigating whether chronic psoriatic inflammation leads to reversible suppression of the hypothalamic–pituitary–gonadal axis, whether stress-related neuroendocrine interactions contribute to flare susceptibility and sex-specific disease trajectories, and whether metabolic dysregulation mediates the link between sex hormone imbalance, Th17-driven inflammation, and keratinocyte hyperproliferation. Addressing these questions in longitudinal cohorts and mechanistic models would help clarify causal relationships and advance an integrated endocrine–immune framework for psoriasis.

In conclusion, sex hormone–psoriasis interactions provide a promising framework for advancing precision medicine in the disease. Continued mechanistic investigation, the incorporation of hormonal parameters into clinical assessment, and the development of multidisciplinary management models will be essential to translating hormonal insights into practical therapeutic benefit, but clinical application should be approached with caution until further evidence supports their reliability.
